# Real-World Experience with CDK4/6 Inhibitors in the First-Line Palliative Setting for HR+/HER2− Advanced Breast Cancer

**DOI:** 10.3390/curroncol32010052

**Published:** 2025-01-20

**Authors:** Ram Patel, John Mathews, Caroline Hamm, Swati Kulkarni, Rasna Gupta, Tarquin Opperman, John Dean Chiong, Abdullah Nasser

**Affiliations:** 1 Schulich School of Medicine and Dentistry, Western University, London, ON N6A 3K7, Canada; topperman2025@meds.uwo.ca (T.O.); jchiong2026@meds.uwo.ca (J.D.C.); abdullah.nasser@wrh.on.ca (A.N.); 2 Department of Medical Oncology, Windsor Regional Hospital, Windsor, ON N8W 1L9, Canada; jmathew4@uwo.ca (J.M.); chamm@uwo.ca (C.H.); skulkar8@uwo.ca (S.K.); rgupta56@uwo.ca (R.G.)

**Keywords:** cyclin-dependent kinase inhibitors, aromatase inhibitors, metastatic breast cancer, survival analysis, real-world evidence

## Abstract

Introduction: CDK4/6 inhibitors in combination with aromatase inhibitors (AIs) are the standard first-line treatment for hormone receptor-positive (HR+), HER2-negative (HER2−) metastatic breast cancer. Landmark trials have demonstrated a comparable progression-free survival (PFS) across CDK4/6 inhibitors, but the overall survival (OS) outcomes have varied. This study aimed to evaluate the real-world PFS and OS for palbociclib and ribociclib when combined with AIs in patients with HR+/HER2− advanced breast cancer. Materials and Methods: This was a retrospective chart review of adult patients with HR+/HER2− metastatic breast cancer treated at a single academic center between 1 January 2015 and 1 December 2022. The baseline demographics, clinical characteristics, and treatment details were extracted. A Kaplan–Meier analysis was used to estimate the PFS and OS, and differences between the treatment groups were assessed using the log-rank test. Cox proportional hazards models were constructed to adjust for confounding factors. Results: Seventy-five patients were included in the final analysis. The cohort was predominantly female (98.7%) and postmenopausal (77.3%), with 52.0% having de novo stage IV disease. Palbociclib was prescribed to 74.7% of the patients, and ribociclib to 25.3%. The patients receiving ribociclib were significantly younger (57.6 vs. 67.5 years, *p* = 0.013) and more likely to be premenopausal (42.1% vs. 5.4%, *p* < 0.001). The real-world median PFS and OS for palbociclib were 20.3 months (95% CI: 14.8–46) and 37.2 months (95% CI: 20.3–not reached [NR]), respectively. For ribociclib, the median PFS and OS were not reached. The Cox proportional hazards models adjusting for age and menopausal status found no significant differences between ribociclib and palbociclib for the PFS (HR = 0.92, *p* = 0.86) or OS (HR = 0.95, *p* = 0.92). Conclusion: In this real-world analysis, palbociclib demonstrated a median PFS consistent with the results from landmark trials, although the observed OS was shorter. The ribociclib-treated patients had a numerically longer PFS and OS compared with those treated with palbociclib, but the differences were not statistically significant. The discontinuation rates were similar between the two groups.

## 1. Introduction

Cyclin-dependent kinase 4 and 6 (CDK4/6) inhibitors have emerged as a cornerstone of treatment for hormone receptor-positive (HR+), human epidermal growth factor receptor 2-negative (HER2−) advanced breast cancer. By selectively targeting the cyclin D-CDK4/6-retinoblastoma protein pathway, these agents effectively halt cancer cell proliferation, enhancing the efficacy of endocrine therapy [1,2,3]. Three CDK4/6 inhibitors, palbociclib, ribociclib, and abemaciclib, have been approved for use in combination with endocrine therapy, and their inclusion in treatment regimens has significantly improved patient outcomes [4,5,6].

Pivotal randomized clinical trials, including PALOMA-2, MONALEESA-2, and MONARCH 2, have demonstrated the superiority of CDK4/6 inhibitors combined with endocrine therapy over endocrine therapy alone in terms of the progression-free survival (PFS) in both the first-line and subsequent treatment settings [7,8,9,10,11,12]. In 2022 and 2023, the median overall survival (OS) data from these trials were published showing a difference between CDK4/6 inhibitors. Palbociclib had a median survival of approximately 54 months and no statistically significant OS difference to endocrine therapy alone. In contrast, both ribociclib and abemaciclib demonstrated a statistically significant median survival greater than 60 months. While these trials have established the clinical benefit of CDK4/6 inhibitors, direct comparisons between these agents are lacking, and the generalizability of the trial results to real-world clinical practice remains uncertain.

In this study, we aimed to assess and compare the real-world PFS and OS outcomes associated with the use of palbociclib and ribociclib in combination with endocrine therapy in patients with HR+, HER2− advanced breast cancer at our cancer center. Our objective was to expand upon the knowledge gained from the pivotal clinical trials and enhance the understanding of the relative effectiveness of these CDK4/6 inhibitors in real-world settings.

## 2. Materials and Methods

### 2.1. Study Design

A retrospective chart review was conducted for patients ≥18 years old with advanced metastatic HR+/HER2− breast cancer at a single academic center diagnosed between 1 January 2015 and 1 December 2022 and treated with first-line palliative AI and CDK4/6 inhibitors. Relevant demographic and clinical variables were extracted and median OS and PFS for each CDK4/6 inhibitor were estimated.

### 2.2. Statistical Analysis

Survival outcomes, including PFS and OS, were analyzed using the Kaplan–Meier method to estimate survival functions. The log-rank test was used to compare survival distributions between treatment groups. Median survival times were reported where at least 50% of patients had experienced the event of interest; otherwise, survival was described as “not reached”.

To adjust for potential confounding factors, multivariable Cox proportional hazards regression models were constructed for both PFS and OS. The models included treatment group, age, PR status, and menopausal status as covariates. Hazard ratios (HRs) and 95% confidence intervals (CIs) were reported to quantify the association between variables and survival outcomes.

All statistical analyses were performed using RStudio (version 2023.12.0+369). The Kaplan–Meier analyses and log-rank tests were conducted using the survival and survminer packages. The Cox proportional hazards models were constructed using the coxph function in the survival package, and survival plots were generated using ggsurvplot to display Kaplan–Meier curves.

### 2.3. Ethics Approval

This study was conducted in accordance with the principles of the Declaration of Helsinki and approved by the institutional ethics review board. As this was a retrospective study, informed consent was waived.

## 3. Results

Of 80 patient charts reviewed, 75 were included in the analysis. Five patients were excluded: one was prescribed abemaciclib, and four did not meet the eligibility criteria due to insufficient survival to initiate treatment or a diagnosis of triple-negative breast cancer. Among the included patients, 98.7% (*n* = 74) were female, with a median age of 63.9 years. The majority were postmenopausal (*n* = 58, 77.3%), while 14.7% (*n* = 11) were premenopausal, and 6.7% (*n* = 5) had an unknown menopausal status. Of the cohort, 52.0% (*n* = 39) had de novo stage IV disease, 72.0% (*n* = 54) were PR-positive, and 65.3% (*n* = 49) were HER2-negative, with a third (33.3%, *n* = 25) classified as HER2-low. Nearly half (42.7%, *n* = 32) had received adjuvant or neoadjuvant chemotherapy. The most prescribed aromatase inhibitor (AI) was letrozole (*n* = 64, 85.3%), followed by anastrozole (*n* = 9, 12.0%) and exemestane (*n* = 2, 2.7%).

Ribociclib was prescribed to 19 patients (25.3%), while 56 patients (74.7%) received palbociclib. The ribociclib group was significantly younger (mean age 57.6 vs. 67.5 years, *p* = 0.013) and more likely to be premenopausal (42.1% vs. 5.4%, *p* < 0.001) compared with the palbociclib group. The baseline characteristics are summarized in Table 1.

**Figure 1:**
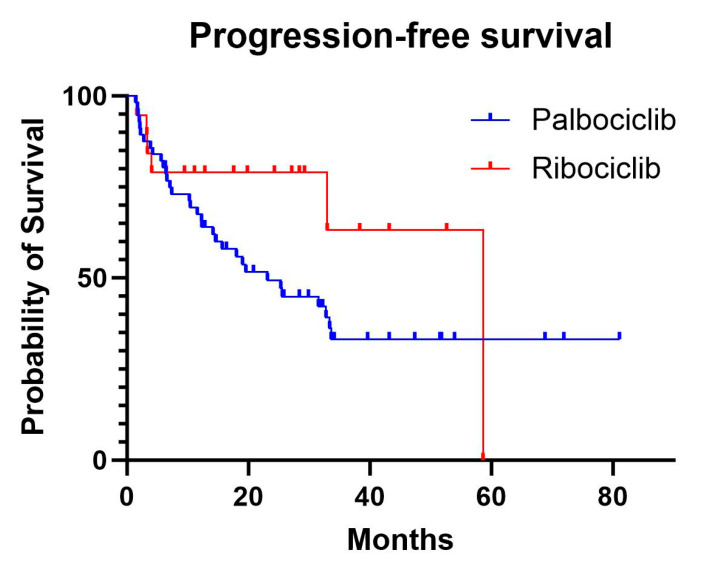
Progression-free survival curves for palbociclib and ribociclib.

The median PFS was 20.3 months in the palbociclib group, while the median was not reached in the ribociclib group, as fewer than 50% of the patients experienced progression (Figure 1). In the Cox proportional hazards model, adjusting for age and menopausal status, there was no significant difference in the PFS between ribociclib and palbociclib (HR, 0.92; 95% confidence interval [CI], 0.35–2.37; *p* = 0.86). Increasing age showed a non-significant trend towards higher progression risk (HR, 1.03; 95% CI, 0.99–1.07; *p* = 0.096), while the premenopausal status had the opposite effect on the progression risk (HR, 0.09; 95% CI, 0.01–1.01; *p* = 0.051).

**Figure 2:**
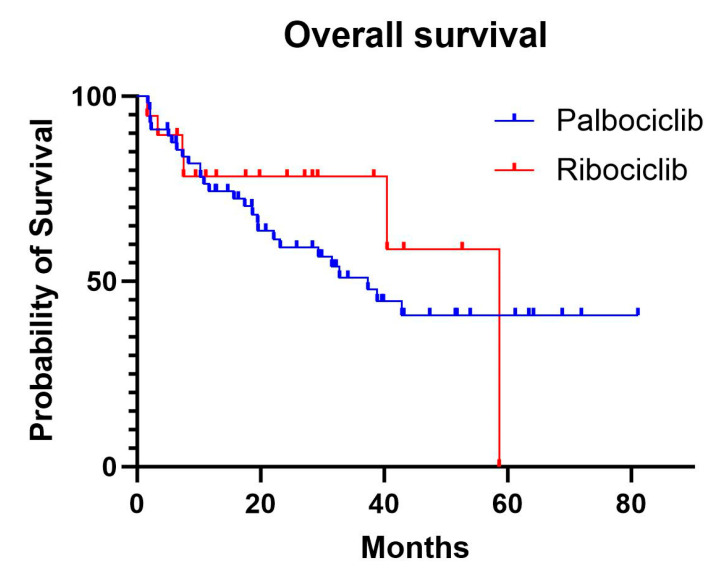
Overall survival curves for palbociclib and ribociclib.

The median OS was 37.2 months in the palbociclib group, while the median was not reached in the ribociclib group due to insufficient events (Figure 2). In the adjusted Cox model, there was no significant difference in the OS between the treatments (HR, 0.95; 95% CI, 0.32–2.80; *p* = 0.92). Age was significantly associated with a worse OS (HR, 1.05; 95% CI, 1.01–1.09; *p* = 0.02). Building on the observed trends in the outcomes with ribociclib and palbociclib, further analysis explored the potential impact of the progesterone receptor (PR) status on survival. Among all the patients, a PR-positive status was associated with a significantly reduced risk of progression compared with PR-negative patients (HR, 0.52; 95% CI, 0.28–0.99; *p* = 0.045). For the OS, a PR-positive status showed a trend toward reduced mortality risk compared with the PR-negative patients (HR, 0.61; 95% CI, 0.30–1.24), though this did not reach statistical significance (*p* = 0.173).

Among the palbociclib group, four (7.14%) patients discontinued the drug due to adverse events. The reasons for discontinuation were neutropenia (two [3.57%]), liver toxicity (one [1.79%]), and renal toxicity (one [1.79%]. Among the ribociclib group, two (10.53%) patients discontinued the agent due to unknown adverse side effects (Table 2).

## 4. Discussion

The introduction of CDK4/6 inhibitors has transformed the treatment paradigm for advanced hormone receptor-positive, HER2-negative breast cancer, establishing them as a cornerstone of first-line therapy. Despite robust evidence from pivotal trials demonstrating their efficacy, direct head-to-head comparisons between these agents in real-world settings remain limited. As a result, the choice of a specific CDK4/6 inhibitor in clinical practice often hinges on considerations such as toxicity profiles, patient comorbidities, and individual tolerability.

In our study, the real-world median PFS for palbociclib was 20.3 months (95% CI: 14.8–46), closely mirroring the 24.8 months (95% CI: 22.1–NR) reported in the PALOMA-2 trial. However, the real-world median OS for palbociclib was 37.2 months (95% CI: 20.3–NR), which is notably shorter than the 53.9 months (95% CI: 49.8–60.8) observed in the trial’s final analysis [2,13]. This discrepancy highlights the challenges of translating trial results into real-world settings, where patients often present with more complex baseline characteristics, a broader spectrum of comorbidities, and less stringent follow-up protocols compared to trial participants.

For ribociclib, the real-world median PFS and OS were not reached due to insufficient events. This reflects the smaller sample size of ribociclib-treated patients in our cohort, a consequence of its more recent introduction compared to palbociclib [14]. Palbociclib’s earlier market approval allowed for greater adoption and a more mature dataset, resulting in a higher number of events for analysis. However, recent trends indicate increasing utilization of ribociclib, suggesting that future studies will provide a more balanced dataset for comparison.

Although statistical significance was not achieved for differences in the PFS and OS between the two agents, ribociclib exhibited a non-significant trend toward improved outcomes. This aligns with data from the MONALEESA-2 trial, where ribociclib demonstrated a median OS of 63.9 months (95% CI: 52.4–71.0), outperforming the 53.9 months (95% CI: 49.8–60.8) observed for palbociclib in the PALOMA-2 trial [15]. While these findings suggest a possible advantage for ribociclib, cross-trial comparisons must be interpreted cautiously due to differences in trial designs, populations, and endpoints.

In our study, there were demographic differences between the two treatment groups. The ribociclib-treated patients were significantly younger (mean age 57.6 vs. 67.5 years; *p* = 0.013) and more likely to be premenopausal (42.1% vs. 5.4%; *p* < 0.001) compared with the palbociclib-treated patients. A younger age and premenopausal status are associated with better resilience and improved survival outcomes in breast cancer [16,17]. This age disparity reflects the clinical practice at our center, where medical oncologists tended to prescribe palbociclib for older patients, prioritizing tolerability over perceived efficacy.

The rates of treatment discontinuation due to adverse events in our cohort were consistent with those reported in the PALOMA-2 and MONALEESA-2 trials. For palbociclib, the real-world discontinuation rate was 7.14%, closely aligning with the 9.7% observed in PALOMA-2 [2,18]. The primary reason for discontinuation was neutropenia, mirroring the trial findings. For ribociclib, the real-world discontinuation rate was 10.53%, slightly higher than the 7.5% reported in MONALEESA-2 [3]. While this suggests comparable tolerability profiles between the two agents, the limited number of ribociclib-treated patients in our cohort precludes definitive conclusions.

A growing body of evidence has sought to compare the efficacy and safety of CDK4/6 inhibitors in the management of HR+/HER2− advanced breast cancer. Kappel et al. (2024) conducted a network meta-analysis assessing the comparative efficacy, safety, and tolerability of abemaciclib, palbociclib, and ribociclib based on data from seven phase 3 randomized controlled trials [16]. When used with an AI backbone, palbociclib demonstrated a numerically, but not statistically, worse OS compared to ribociclib and abemaciclib. No significant OS differences were observed between ribociclib and abemaciclib. In combination with fulvestrant, palbociclib had a comparable OS to that of ribociclib and abemaciclib, with no significant differences between ribociclib and abemaciclib. In terms of safety, abemaciclib was associated with higher rates of gastrointestinal toxicity and infections but lower rates of neutropenia compared to palbociclib. Ribociclib demonstrated higher incidences of QT prolongation, transaminitis, and gastrointestinal side effects relative to palbociclib. Notably, abemaciclib had significantly higher treatment discontinuation rates due to adverse events compared to both ribociclib and palbociclib, as well as a significantly higher rate of treatment-related deaths versus ribociclib, with a numerically but non-significant increase compared to palbociclib.

Al-Ziftawi et al. (2023) conducted a real-world study in Qatar, comparing the effectiveness and safety of palbociclib and ribociclib in 108 patients [17]. No significant differences were observed in the progression-free survival (PFS; 17.85 vs. 13.55 months) or OS (29.8 vs. 31.7 months) between the two groups. Ribociclib was associated with a significantly higher rate of QT prolongation compared to palbociclib.

Dajsakdipon et al. (2024) provided additional insights with a retrospective cohort study in Thailand involving 183 patients treated with first-line AI plus ribociclib or palbociclib. This study highlighted a non-significant trend toward a longer median OS with palbociclib compared to ribociclib [19]. Differences in the OS might reflect variations in demographics, genetic factors, and healthcare access between Western and Asian populations. The subgroup analysis suggested a potential OS benefit with ribociclib in patients with ≥3 metastatic sites or coronary artery disease. The median PFS was comparable between the two groups (31.8 months for palbociclib vs. 27.9 months for ribociclib). Regarding toxicity, ribociclib had lower rates of neutropenia and thrombocytopenia but required more frequent dose adjustments, while both agents had similar rates of dose reductions and time to dose reduction. These studies collectively highlight differences in efficacy and safety profiles among CDK4/6 inhibitors, which may guide personalized treatment decisions for patients with HR+/HER2− advanced breast cancer.

## 5. Conclusions

In conclusion, this study highlights the need for further real-world research to better delineate the relative effectiveness of ribociclib and palbociclib. Larger, multicenter datasets with balanced cohorts and a longer follow-up are essential to validate these findings and address the limitations of smaller sample sizes and demographic heterogeneity.

## Figures and Tables

**Table 1:** Patient characteristics and aromatase inhibitor use.

	Palbociclib (*n* = 56)	Ribociclib (*n* = 19)
Female	55 (98.21%)	19 (100%)
Median age (IQR)	66.69 (59.78–75.18)	52.14 (49.12–67.02)
Postmenopausal	50 (89.29%)	8 (42.11%)
Premenopausal	3 (5.36%)	8 (42.11%)
De novo presentation	31 (55.36%)	11 (57.89%)
Grade I	11 (19.64%)	5 (26.32%)
Grade II	28 (50.00%)	7 (36.84%)
Grade III	12 (21.43%)	6 (31.58%)
Positive PR status	38 (67.86%)	16 (84.21%)
Negative PR status	17 (30.36%)	3 (15.79%)
HER2-negative (IHC 0)	36 (64.29%)	13 (68.42%)
HER2-low (IHC 1+/2+, negative FISH)	19 (33.93%)	6 (31.58%)
Received chemotherapy in past	24 (42.86%)	8 (42.11%)
Letrozole	49 (87.50%)	15 (78.90%)
Anastrozole	5 (8.90%)	4 (21.1%)
Exemestane	2 (3.60%)	0 (0.00%)

**Table 2:** Rates of discontinuation for palbociclib and ribociclib due to adverse side effects.

	Palbociclib (*n* = 56)	Ribociclib (*n* = 19)
Overall	4 (7.14%)	2 (10.53%)
Neutropenia	2 (3.57%)	0
Hepatic toxicity	1 (1.79%)	0
Renal toxicity	1 (1.79%)	0
Unknown side effects	0	2 (10.53%)

## Data Availability

Anonymized data can be made available by contacting the senior author.
